# Hand Knitting Induced Thrombosis of the Subclavian Vein in a Young Woman: an Unusual Cause of Paget-Schroetter Syndrome

**DOI:** 10.4084/MJHID.2011.022

**Published:** 2011-05-16

**Authors:** Aytekin Alcelik, Haluk Savli, Ahsen Zeyrek, Abdullah Yalcin

**Affiliations:** Deparment of Internal Medicine, Abant Izzet Baysal University, Bolu, Turkey

## Dear Editor,

Effort thrombosis refers to axillary-subclavian vein thrombosis associated with strenuous and repetitive activity of the upper extremities. Spontaneous thrombosis of upper extremity veins, subsequently termed the Paget-Schroetter syndrome, is relatively rare.^1^ Axillary vein thrombosis is associated with various aetiological factors. Early recognition and aggressive treatment of this disorder has been recommended to avoid the long-term sequelae associated with chronic venous obstruction.^2^ Effort thrombosis usually follows sporting activities, such as wrestling, playing ball, gymnastics and swimming, which involve vigorous and sustained upper extremity movements.^3^ As a common leisure activity of Turkish women, hand knitting was not previously reported as a cause of upper extremity thrombosis. We report what to our knowledge is the first case of effort thrombosis of an upper extremity caused by hand knitting.

### Case:

A 26 -year-old woman presented with progressive left arm swelling that had started one week before admission. Her past medical history was unremarkable for trauma to the shoulder or arm, venous catheter insertion or intravenous drug usage. She was otherwise healthy and did not report any personal or family history of hematological disorders and denied using oral contraceptives. On further questioning, the patient gave a history of knitting daily, at continuous stretches of 4–6 hours. Physical examination revealed nonpitting edema involving the entire left upper extremity. The left upper limb showed normal arterial pulses and there was no neurological deficit or bony injury. A color Doppler and duplex ultrasound scan of the left upper limb showed acute deep vein thrombosis involving the left axillary and subclavian veins.

Blood was taken for factor V Leiden-factor II (G20210A) mutations and antinuclear antibody concentrations, all of which were normal. The coagulation profile revealed activated partial thromboplastin time and prothrombin time within normal range. Anticoagulation was then started with heparin and continued with warfarin. As an outpatient, she completed 8 weeks of anticoagulation therapy. A repeat ultrasound examination showed that the thrombus had resolved completely and the left subclavian vein appeared intact. After having excluded other possible predisposing factors for upper limb deep vein thrombosis, we concluded that our patient`s thrombosis was primarily prolonged knitting induced.

### Discussion:

The upper limb is an uncommon site for deep vein thrombosis. Primary axillary-subclavian vein thrombosis is, however, well described and is also called Paget-Schroetter syndrome.^4^ Clinical presentation of major venous thrombosis in the upper limb usually presents with swelling of the upper limb, prominence of superficial veins and neurological symptoms.^5^ The diagnosis of effort thrombosis should be suspected clinically and confirmed with contrast venography or, as in this case, duplex ultrasonography.^6^

In 1894, von Schroetter was the first to identify vascular trauma from muscle strain as a potential etiologic factor. It is believed that retroversion, hyperabduction and extension of the arm involved with these activities impose undue strain on the subclavian vein leading to microtrauma of the endothelium and activation of the coagulation cascade. Substantial evidence now supports the role of anatomical abnormalities involving the thoracic outlet in the pathogenesis of effort thrombosis.^3^,^7^

Effort thrombosis of the upper extremity has been described in athletes involved in a wide variety of sports, including ball games, combatant sport and heavy athletics, games with rackets or clubs, push-up exercise and aquatic sports.^3^,^8^ Phipps et al asserted that portable computer gaming can potentially produce the same degree of repetitive muscular activity and associated trauma and compression may cause venous thrombosis.^9^ Additionally, Snead et al theorized that this repeated action could be the mechanism of venous intimal injury and subsequent axillosubclavian venous thrombosis in the absence of an identifiable external compression.^10^ We believe that hand knitting may also cause venous thrombosis with the same mechanisms, namely, by repetitive minor injuries.

### Conclusions:

Hand knitting might be a potential risk for upper limb thrombosis. Effort thrombosis should be suspected in women who perform repeated hand knitting and present with symptoms characteristic of effort thrombosis.
